# Gender moderates the association between resting vagally mediated heart rate variability and attentional control

**DOI:** 10.3389/fpsyt.2023.1165467

**Published:** 2023-08-16

**Authors:** Xiaocong Zhang, Siyu Wang, Yueyi Sun, Yanwen Ding

**Affiliations:** Department of Psychology, School of Medicine and Holistic Integrated Medicine, Nanjing University of Chinese Medicine, Nanjing, Jiangsu, China

**Keywords:** attentional control, heart rate variability, gender differences, vagal tone, moderating effects

## Abstract

**Background:**

Women typically exhibit weaker attentional control ability than men. Lower resting vagally mediated heart rate variability (vmHRV) is thought to reflect the poorer function of the neurophysiological pathways underlying attentional control and thus, poorer attentional control ability. However, existing findings are inconsistent regarding the relationship between vmHRV and attentional control. Gender may be an important moderator.

**Objective:**

To examine whether gender moderates the relationship between resting vmHRV and attentional control, and to provide neurophysiological evidence for elucidating gender differences in attentional control ability.

**Methods:**

Two hundred and twenty college students completed the Attentional Control Scale to evaluate their attentional control ability. Resting vmHRV was assessed during a 5 min baseline period using an electrocardiographic amplifier (ECG100C) of the Biopac MP150 physiological recorder.

**Results:**

(1) There was no significant difference in the total scores of the Attentional Control Scale between men and women (*t* = 0.498, *p* > 0.05), but the scores of the attentional shifting dimension of women were significantly lower than those of men (*t* = 1.995, *p* < 0.05); (2) Resting vmHRV was significantly negatively correlated with attentional control in women(*r* = −0.233, *p* < 0.01), whereas the correlation was not significant in men; (3) Gender significantly moderated the relationship between resting vmHRV and attentional control (*B* = −3.088, 95% boot CI [−5.431, −0.745], *t* = −2.598, *p* < 0.05); (4) Among participants with lower resting vmHRV, there was no significant difference in attentional control between men and women (*B* = 2.284, 95% boot CI [−0.748, 5.310], *p* > 0.05), but among participants with higher resting vmHRV, men scored significantly higher than women in attentional control (*B* = −3.377, 95% boot CI [−6.406, −0.348], *p* < 0.05).

**Conclusion:**

Gender moderates the relationship between resting vmHRV and attentional control, with higher resting vmHRV in women reflecting a compensatory response to deficits in attentional control.

## 1. Introduction

Attentional control is a limited capacity system that allows individuals to actively control their attention and is an important component of top-down self-regulation (1). Attentional control plays an important role in emotion regulation, helping individuals select optimal responses based on situational needs and suppress less functional responses (2). Attentional control includes two dimensions such as attentional focusing and attentional shifting (3), which help individuals prevent and reduce depression (4, 5). Attentional focusing refers to an individual’s ability to maintain attentional focusing, helping individuals effectively allocate attentional resources to target information when faced with competing or conflicting information (6). Attentional focusing also helps to suppress attentional bias toward threatening stimuli, attenuate negative emotionally driven responses, and increase attention to positive information and ideas (7). Attentional shifting, which refers to an individual’s ability to shift attention from one stimulus or task to another, helps individuals to divert attentional resources away from negative information and ideas, thereby avoiding ruminative thinking (8). Evidence from behavior and neuroimaging studies suggests that deficits in attentional control are important factors in the development of depressive mood (9).

According to the neurovisceral integration model, attentional control is significantly influenced by prefrontal-subcortical inhibitory pathways (10). The prefrontal cortex and cingulate gyrus are important brain structures involved in attentional control. The prefrontal cortex exerts inhibitory control over sympathoexcitatory subcortical circuits, including the amygdala (2, 11), allowing individuals to respond in a manner appropriate to the demands of different environments. Reduced prefrontal regulation can result in overactive subcortical activity, triggering responses such as hypervigilance, worry, and rumination (12).

Heart rate variability (HRV) is a measure of the variability of the interval between two consecutive heartbeats (13). Vagally mediated HRV (vmHRV) can demonstrate top-down active attentional control in an individual (14), which can be influenced by the central brain networks involved in attentional regulation, especially the prefrontal cortex. Neurobiological studies have shown that the neural network comprising the prefrontal cortex, limbic system, and brainstem rhythmically regulates the cardiac sinus node via the vagus nerve, thereby influencing heart rate variability (9, 15). Lower resting vmHRV is associated with hyporegulation of the prefrontal cortex, resulting in overactive subcortical structures that prevent individuals from exercising effective attentional control (12). Individuals with lower resting vmHRV are prone to being constantly hypervigilant to the environment and cannot effectively inhibit the interference of negative stimuli, which can easily lead to worry and tension (2, 16). Individuals with higher resting vmHRV levels show stronger attentional control. They can efficiently direct attentional resources toward information that is relevant to the activity at hand, minimize interference from irrelevant information, and improve the effectiveness of cognitive processing (16). Additionally, people with higher resting vmHRV are better able to divert their attention away from unpleasant emotional cues, which reduces ruminative thinking (17).

However, there are inconsistent findings in existing studies regarding the relationship between resting vmHRV and attentional control ability. Although some studies have shown that resting vmHRV is positively correlated with attentional control (16, 17), others have shown that the correlation is not significant (14). Therefore, we speculate that certain factors may influence the relationship between resting vmHRV and attentional control. Gender may be an important moderator.

Studies have shown that there are gender differences in attentional control (18, 19). Women are weaker than men at focusing attention and are more easily distracted by information that is not relevant to the task at hand (20). Women are also more likely to be attracted to emotionally distracting stimuli that impair cognitive processing performance (21). In addition, women have weaker attentional shifting abilities than men and are less efficient at shifting attention between multiple cognitive processing tasks (18). Gender differences in attentional control abilities may help explain why women are more vulnerable to emotional problems such as depression (22).

The differences in attentional control between men and women have a neurophysiological basis. The brain regions involved in attentional control mainly include structures such as the frontal cortex and cingulate gyrus (9). Neurostructurally, women have a larger anterior cingulate gyrus and frontal cortex (including the prefrontal cortex) than men (23). From a neurofunctional perspective, women have higher levels of activation in the anterior cingulate and amygdala than men when faced with negative stimuli (24), suggesting that women are more likely to be captured by negative information and produce more negative emotional experiences. In addition, women also have significantly higher levels of prefrontal activation than men, suggesting that women make greater efforts to cope with unpleasant emotions (25). There are also gender differences in the corticotropin-releasing factor (CRF) receptors, which are related to the stress response. The locus coeruleus arousal system in women is less adaptable to CRF hypersecretion in the face of negative stimuli, which impairs women’s attentional control and leads to hypervigilance in response to environmental stimuli, resulting in women being more prone to depression (22).

A growing body of research has shown that gender is a significant factor in moderating the relationship between resting vmHRV and mood, such as depression, anxiety and stress perception. For example, there are gender differences in the relationship between resting vmHRV and depression (26). Higher resting vmHRV is shown in highly depressed women compared to less depressed women, while lower resting vmHRV is seen in highly depressed men compared to less depressed men (27). Studies on adult cynomolgus monkeys have also found that depressed females show higher resting vmHRV than non-depressed females (28). There is growing evidence that high resting vmHRV levels in women and men may reflect different mechanisms of emotion regulation (29, 30). Neuroimaging studies have shown that resting vmHRV levels in men are associated with reduced amygdala activity, whereas resting vmHRV levels in women are associated with increased amygdala activity (31, 32). Furthermore, the degree of prefrontal activity was positively associated with depression levels, reflecting a compensatory effect of prefrontal activity on depressed mood (26). Researchers hypothesized that higher resting vmHRV in men reflects a tonic inhibition of prefrontal activity in subcortical threat response circuits, which reduces adverse emotions such as depression; whereas higher resting vmHRV in women reflects a compensatory mechanism of prefrontal activity in response to subcortical threat response circuits, indicating that women make more emotional regulation efforts to cope with negative emotions (29, 30).

In summary, gender may be an important factors that moderates the relationship between resting vmHRV and attentional control. However, existing studies have not investigated this issue systematically. The current study aimed to examine how gender moderates the relationship between resting vmHRV and attentional control to shed light on the neurophysiological differences in attentional control between men and women.

## 2. Materials and methods

### 2.1. Participants

Participants were recruited through two methods. One group of participants was recruited through campus advertisements and received a cash incentive of 15 RMB for participation. The other group of participants was recruited from students enrolled in public psychology courses and received a course credit award. Inclusion criteria included being right-handed and having normal or corrected-to-normal vision. Exclusion criteria included a history of neurological, psychiatric or cardiovascular disease. A total of 220 participants (96 male and 124 female) were included in this study, with a mean age of 19.31 ± 1.17 years. All participants voluntarily signed the informed consent form before the experiment. As sleep quality, physical activity and caffeine intake are important variables influencing HRV (33), participants were asked to get enough sleep the night before the experiment and to refrain from vigorous physical activity and caffeine intake in the 6 h before the experiment.

### 2.2. Experimental flow

When participants arrived at the laboratory, they naturally sat on a chair to rest, signed the experimental informed consent form, and filled out a series of self-report questionnaires. After the participants felt completely relaxed, they began to collect resting ECG data for 5 min. During the ECG data acquisition process, the participants sat relaxed in a chair, kept their bodies stable, breathed naturally, and looked at the picture of the neutral cup presented on the computer screen. At the end of experiment, participants scored their emotional arousal on a 9-point scale.

### 2.3. Attentional control scale

The Attentional Control Scale (ACS) is a self-reported measure of attentional control ability (1). It is widely used in clinical and experimental research as a convenient and valid measure of attentional control ability (34). The scale consists of 20 items and is scored on a 4-point scale (1 = almost never, 4 = almost always), with higher scores indicating better attentional control. The scale can be divided into two subdimensions: attentional focusing (e.g., “I have difficulty focusing on a difficult task when there is noise around me”) and attentional shifting (e.g., “I can switch quickly from one task to another”). In this study, the scale’s internal consistency coefficient is 0.80.

### 2.4. Emotional arousal report

To assess the subjective emotional experience during the experiment, we instructed the participants to score their emotional arousal (1 refers to extremely calm, 9 refers to extremely nervous) on a 9-point scale (35).

### 2.5. Resting vmHRV measurement

Cardiac data were collected using the electrocardiographic amplifier (ECG100C) of the Biopac MP150 physiological recorder. Three silver chloride (Ag-AgCL) disposable electrode pads were attached to the participant’s wrist and two ankles in lead II. The filtering range of the Biopac ECG100C amplifier was 0.5–35 Hz with a sampling rate of 1,000 Hz. The Kubios HRV analysis package (36) was used to filter the ECG data offline, remove artifacts, and calculate the root mean square of successive heartbeat interval differences (RMSSD). RMSSD is the recommended parameter for field studies, as it is less affected by respiration and body movement and is more reliable than high frequency (HF) HRV (37). We therefore employed RMSSD as our primary metric of vmHRV. RMSSD values were natural log transformed (ln) to obtain normally distributed data. The heart rate and respiratory rate of the participants were also extracted from the ECG data in this study. Resting HRV is known to decrease with age and body mass index (BMI) and may be influenced by the participant’s respiration and emotional arousal (29, 35, 38, 39). Therefore, age, BMI, respiratory rate and emotional arousal were included as covariates.

### 2.6. Statistical analyses

All statistical analysis was conducted using SPSS 26.0 (IBM Chicago, IL). All tests were two-tailed and significance levels were evaluated using an alpha of 0.05. Independent samples *t*-tests were conducted to analyze the differences between male and female participants on all variables. Pearson correlation was used to analyze the relationship between vmHRV and other important variables. The SPSS macro PROCESS (Model 1) was used to test the moderating effect of gender (moderator; 1 = men, 2 = women) on the relationship between attentional control (independent variable) and resting vmHRV (dependent variable) (40). Bootstrap repeated sampling 5,000 times to obtain the parameter estimation and 95% confidence interval.

## 3. Results

Table 1 presents the results of the gender difference test for several important variables. Male participants had a significantly higher BMI than female participants (*t* = 4.133, *p* < 0.001). There was no significant difference between male and female participants on resting vmHRV (*t* = −0.002, *p* = 0.998). Women’s attentional control scores were slightly lower than men’s, with the difference not reaching a significant level (*t* = 0.498, *p* = 0.619). However, women scored significantly lower than men on the attentional shifting dimension of attentional control (*t* = 1.995, *p* < 0.05).

**Table 1:** Independent samples *t*-test for relevant indicators for male and female participants (M ± SD).

	Men (*N* = 96)	Women (*N* = 124)	*t*	*p*	Cohen’s *d*
Age	19.16 ± 1.01	19.43 ± 1.28	−1.709	0.089	−0.232
BMI	23.16 ± 5.26	20.65 ± 3.71	4.133	0.000	0.562
Emotional Arousal	3.58 ± 1.33	3.41 ± 1.15	1.026	0.306	0.137
Resting vmHRV	3.69 ± 0.91	3.69 ± 0.92	−0.002	0.998	0.000
Respiration	0.26 ± 0.06	0.27 ± 0.06	−1.286	0.200	−0.175
Mean HR	82.14 ± 13.71	83.46 ± 11.57	−0.773	0.440	−0.105
Total ACS	51.43 ± 7.69	50.88 ± 8.40	0.498	0.619	0.068
Attentional focusing	25.80 ± 4.47	26.39 ± 5.06	−0.894	0.372	−0.122
Attentional shifting	25.63 ± 4.12	24.49 ± 4.22	1.995	0.047	0.271

Age was measured in years; body mass index (BMI) was measured in kg/m^2^; emotional arousal represents the subjective emotional experience of participants during the experiment; resting vmHRV is represented as the natural log transform of root mean square of successive heartbeat interval differences (RMSSD) values, and respiration is indexed using high-frequency HRV peak values; mean HR (heart rate) is in beats per minute. Total ACS represents the total score on the Attentional Control Scale (ACS); attentional focusing and attentional shifting represent scores on the 2 sub-dimensions of the ACS scale.

Table 2 shows the correlations among variables of interest in both the entire sample and split by gender. No significant correlation was observed between vmHRV and total attentional control scores in both the whole sample and the male sample. When the female sample was analyzed separately, a significant negative correlation between resting vmHRV and attentional control was observed (*r* = −0.233, *p* < 0.01).

**Table 2:** Correlation analysis results for the entire sample and split by gender.

(a) All participants	1	2	3	4	5	6	7	8
1. Resting vmHRV	—							
2. ACS total	−0.090	—						
3. Gender	0.001	−0.034	—					
4. Age	0.065	−0.075	0.115	—				
5. BMI	0.105	−0.045	−0.270^**^	−0.076	—			
6. Respiration	−0.110	−0.077	0.087	0.037	−0.030	—		
7. Mean HR	−0.374^**^	0.045	0.052	−0.097	−0.056	0.138^*^	—	
8. Emotional Arousal	0.045	0.135^*^	−0.069	−0.044	−0.025	−0.097	0.008	—

(b) Men	1	2	3	4	5	6	7
1. Resting vmHRV	—						
2. ACS total	0.115	—					
3. Age	−0.116	0.039	—				
4. BMI	0.161	−0.039	0.017	—			
5. Respiration	−0.194	−0.152	0.130	−0.030	—		
6. Mean HR	−0.419^**^	−0.070	−0.008	−0.116	0.160	—	
7. Emotional Arousal	0.081	0.111	−0.202^*^	0.035	−0.026	−0.043	—

(c) Women	1	2	3	4	5	6	7
1. Resting vmHRV	—						
2. ACS total	−0.233^**^	—					
3. Age	0.175	−0.133	—				
4. BMI	0.057	−0.077	−0.107	—			
5. Respiration	−0.045	−0.020	−0.036	0.018	—		
6. Mean HR	−0.336^**^	0.146	−0.176^*^	0.052	0.110	—	
7. Emotional Arousal	0.015	0.153	0.078	−0.148	−0.151	−0.022	—

^*^Represents *p* < 0.05 and ^**^represents *p* < 0.01.

**Figure 1:**
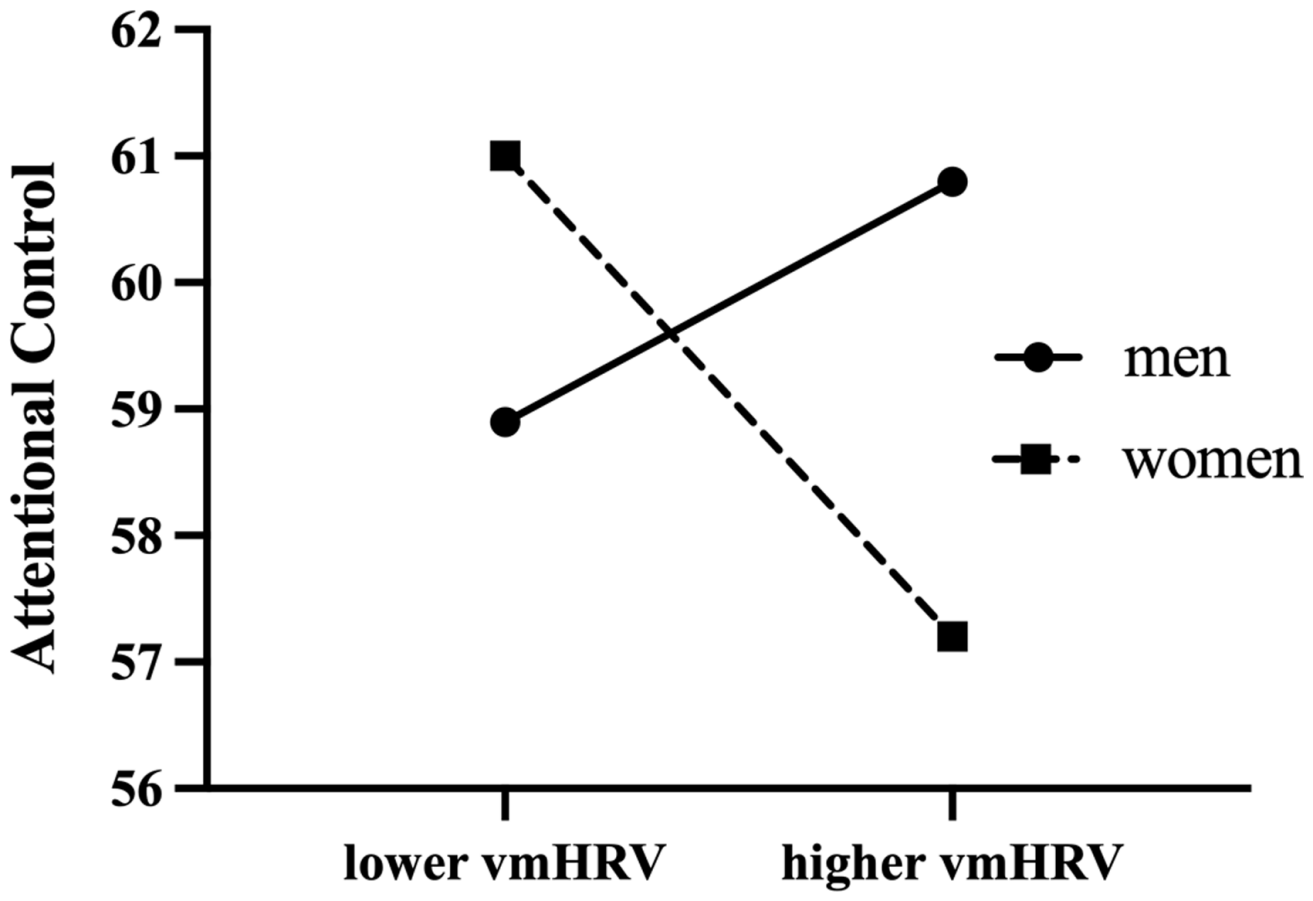
Moderating effects of gender on resting vmHRV and attentional control.

Table 3 presents the moderation analyses of prediction of attentional control ability. Analysis of moderation effects showed that gender significantly moderated the relationship between resting vmHRV and attentional control (*B* = −3.088(1.189), 95% boot CI [−5.431, −0.745], *t* = −2.598, *p* = 0.01). Conditional analysis showed a negative correlation between resting vmHRV and attentional control in women (*B* = −2.120 (0.779), 95% boot CI [−3.656, −0.585], *p* = 0.007), whereas there was no significant correlation between resting vmHRV and attentional control in men (*B* = 0.968 (0.898), 95% boot CI [−0.802, 2.737], *p* = 0.282). The moderating effect of gender remained significant when analyzed with age, BMI, respiration and emotional arousal as covariates in the moderation analysis (*B* = −2.897(1.203), 95% boot CI [−5.268, −0.526], *t* = −2.408 *p* = 0.017), with a negative correlation between resting vmHRV and attentional control in women (*B* = −2.072 (0.786), 95% boot CI [−3.621, −0.523], *p* = 0.001) and no significant correlation in men (*B* = 0.825 (0.912), 95% boot CI [−0.973, 2.622], *p* = 0.367). In other words, among women, the difference in attentional control between participants with lower and higher resting vmHRV was significant; whereas among men, the difference in attentional control between participants with lower and higher resting vmHRV was not significant (see Figure 1). With gender as the independent variable and resting vmHRV as the moderating variable (reversing the independent and moderating variables for the above analysis), the moderating effect remained significant. Conditional analysis showed no significant difference in attentional control between men and women in participants with lower resting vmHRV (*B* = 2.284 (1.542), 95% boot CI [−0.748, 5.310], *p* = 0.139), but men scored significantly higher in attentional control than women in participants with higher resting vmHRV (*B* = −3.377 (1.536), 95% boot CI [−6.406, −0.348], *p* = 0.029).

**Table 3:** Moderation analyses of gender on resting vmHRV and attentional control.

	Model 1				Model 2			
	*B*	SE	95% CI	*t*	*B*	SE	95% CI	*t*
Resting vmHRV × Gender	−3.088	1.189	[−5.431, −0.745]	−2.598^*^	−2.897	1.203	[−5.268, −0.526]	−2.408^*^
Resting vmHRV	4.056	1.957	[22.372, 51.671]	2.072^*^	3.722	1.985	[−0.191, 7.634]	1.875
Gender	10.838	4.515	[1.939, 19.737]	2.401^*^	10.180	4.532	[1.246, 19.114]	2.461^*^
Age					−0.265	0.468	[−1.187, 0.657]	−0.567
BMI					−0.099	0.122	[−0.340, 0.142]	−0.813
Respiration					−8.214	9.065	[−26.083, 9.654]	−0.906
Emotional Arousal					0.799	0.440	[−0.068, 1.666]	1.817
	*F* = 2.942, *p* = 0.034, *R*^2^ = 0.039				*F* = 2.080, *p* = 0.047, *R*^2^ = 0.064			

*B*, unstandardized beta coefficients; SE, standard errors; 95%CI, 95% bootstrapping confidence intervals; Resting vmHRV × Gender represents the interaction term between resting vmHRV and gender; gender was coded as ‘1’ for men and ‘2’ for women. In model 2, age, BMI, respiratory and emotional arousal were added as covariates to predict attentional control. ^*^Represents *p* < 0.05.

## 4. Discussion

This study aimed to investigate whether gender moderates the relationship between resting vmHRV and attentional control, and to provide neurophysiological evidence for elucidating gender differences in attentional control ability. The results showed that there was no significant difference between men and women in the overall scores of attentional control, but women scored significantly lower than men in attentional shifting. Gender was a significant variable in moderating the relationship between resting vmHRV and attentional control. Resting vmHRV was significantly negatively correlated with attentional control in women, whereas it was not significantly correlated with attentional control in men.

Although there was no significant difference in the total scores of attentional control between men and women in this study, women had poorer attentional shifting abilities than men, which confirmed the previous findings. Women had weaker attentional shifting ability than men, as evidenced by a lower efficiency in shifting attention across multiple cognitive processing tasks (18). Attentional shifting helps individuals shift their attention away from negative information and thoughts and facilitates the reduction of ruminative thinking (5, 7). As ruminative thinking is one of the core causes of depressed mood (4, 8, 9), the results of the present study help to explain why women are more likely to suffer from depression.

More importantly, the present study showed that gender moderates the relationship between resting vmHRV and attentional control, with resting vmHRV negatively correlated with attentional control in women, whereas the two were not significantly correlated in men. The conditional analysis revealed that among participants with higher resting vmHRV, men had significantly higher attentional control scores than women. This result explains the mechanism of gender differences in attentional control from the perspective of vagal nerve tone. Women with high resting vmHRV were more likely to have difficulty with attentional control, suggesting differences in the neural mechanisms of attentional control reflected by high resting vmHRV in men and women. Consistent with previous studies, we speculated that higher vmHRV in female participants reflects a compensatory mechanism for deficits in attentional control, as an effort by individuals to compensate for dealing with negative emotional responses (29, 30). Due to their inability to effectively block the interference from negative information and thoughts, and to effectively shift their attention away from negative information and thoughts, women with weaker attentional control abilities are more likely to experience overactive subcortical sympathetic excitatory cortex, which requires higher prefrontal activity for a compensatory response, thus exhibiting higher vmHRV levels (26, 30). The vagal tank theory states that cardiac vagal control serves as an indicator of the efficiency of mobilization and use of self-regulatory resources (41). Relatively high levels of resting vmHRV indices imply greater psychophysiological resources (e.g., integration of neural, metabolic, and cognitive resources) available for self-regulation, resulting in adaptive emotional responses. High resting vmHRV in women may represent a greater reserve of self-regulatory resources which are needed in individuals with low attentional control and high depression.

Some studies support the idea that higher resting vmHRV in women is a compensatory response to inadequate attentional control. Studies have shown that higher resting vmHRV in women is associated with increased activity in the amygdala (the area associated with threat processing), whereas higher resting vmHRV in men is associated with decreased amygdala activity (31). This compensatory response to threats and depressive symptoms has a neural basis in the prefrontal cortex. Studies have shown that major depressive episodes are associated with greater medial prefrontal activity (26). Women with poorer attentional control are prone to frequent and sensitive awareness of negative emotions (7, 9), and thus tend to show more depressive symptoms (28). On a neurophysiological level, they would show great amygdala activity, which requires stronger vagal activity (greater prefrontal activity) to compensate (2, 11, 12, 15), resulting in increased HRV. Thus, high resting vmHRV in women is associated with inadequate attentional control. Taken together, higher resting vmHRV may be important for women to maintain psychological wellbeing. In recent years, HRV biofeedback (HRV-BF) has received considerable attention because of its effectiveness in modifying HRV and its positive influence on attentional control and emotions (41). In future clinical work, healthcare professionals could use HRV-BF interventions with women who experience difficulties with attentional control to address mood problems such as depression.

From an evolutionary psychology perspective, the tend-and-befriend theory and the parental investment theory could also provide an explanation for the negative correlation between resting vmHRV and attentional control is limited to women (38, 42). Evolutionary psychological theory and neurophysiological evidence suggest that males are more likely to adopt a “fight or flight” response mode when faced with environmental stress and danger, showing a higher vagal withdrawal response (42). Women tend to respond to stress with behavioral, neuroendocrine, and autonomic responses that support the maintenance and acquisition of social bonds. They tend to delay impulsivity for the benefit of their offspring, and their response patterns include greater efforts at attentional control and emotional regulation, showing higher vagal activity (38). These responses are beneficial for women to compensate for high levels of distress and promote intimacy. These views are consistent with the neurovisceral integration model which advocates that vmHRV reflects attentional and emotion regulation abilities (12, 43). Relative to men, higher resting vmHRV in women may reflect a compensatory mechanism of greater attentional control and emotion regulation efforts in response to adverse emotions. In contrast, higher resting vmHRV in men may represent lower maladaptive emotions achieved through tonic inhibition of subcortical threat circuits (44).

In general, the results of this study suggest that women have poorer attentional shifting abilities than men, and that gender moderates the relationship between vmHRV and attentional control. The high resting vmHRV levels in men and women may reflect different emotion regulation mechanisms, with higher vmHRV in women reflecting a compensatory response to deficits in attentional control.

## 5. Limitations

There are several shortcomings in the present study. First, the present study examined the relationship between resting vmHRV and attentional control in a cross-sectional investigation and was unable to determine a causal relationship between the two variables. Future studies could design longitudinal follow-up experiments to examine the neurophysiological basis of gender differences in attentional control. Second, the present study only used the self-report scale to measure attentional control ability, which may not accurately cover all aspects of attentional control (1). Future research could combine self-report scales with psychological experimental measures (e.g., Stroop task, Flanker task, eye-tracking experiments, etc.) to provide a more comprehensive assessment of attentional control ability. Third, recent research has suggested that the menstrual cycle in women may influence the relationship between vmHRV and mental status (45). Future studies should control for the menstrual cycle phase of female participants to better investigate the relationship between resting vmHRV and attentional control. Fourth, the study only examined a sample of healthy young adults, and the findings may not be applicable to other age groups or individuals with medical conditions. Future studies could investigate the relationship between vmHRV and attentional control in a broader population.

## Data availability statement

The original contributions presented in the study are included in the article/supplementary material, further inquiries can be directed to the corresponding author.

## Ethics statement

The studies involving human participants were reviewed and approved by Ethics Committee of Nanjing University of Chinese Medicine. The patients/participants provided their written informed consent to participate in this study.

## Author contributions

XZ contributed to the conception and design of the study. YS checked and corrected the scientific issue of the study. XZ and SW conducted the experiment, analyzed the data, and wrote the first draft of the manuscript. All authors contributed to the article and approved the submitted version.

## Funding

This research was funded by the Research Project of Jiangsu University Philosophy and Social Science (2021SJA0338).

## Conflict of interest

The authors declare that the research was conducted in the absence of any commercial or financial relationships that could be construed as a potential conflict of interest.

## Publisher’s note

All claims expressed in this article are solely those of the authors and do not necessarily represent those of their affiliated organizations, or those of the publisher, the editors and the reviewers. Any product that may be evaluated in this article, or claim that may be made by its manufacturer, is not guaranteed or endorsed by the publisher.
